# A Longitudinal Study of Plasma Glycated Albumin across Pregnancy and Associations with Maternal Characteristics and Cardiometabolic Biomarkers

**DOI:** 10.1093/clinchem/hvad172

**Published:** 2023-11-02

**Authors:** Wei Wei Pang, Stefanie N Hinkle, Jing Wu, Paulina Stallcup, Michael Y Tsai, David B Sacks, Cuilin Zhang

**Affiliations:** Global Center for Asian Women’s Health (GloW), Yong Loo Lin School of Medicine, National University of Singapore, Singapore; Bia-Echo Asia Centre for Reproductive Longevity & Equality (ACRLE), Yong Loo Lin School of Medicine, National University of Singapore, Singapore; Department of Obstetrics and Gynaecology, Yong Loo Lin School of Medicine, National University of Singapore, Singapore; Department of Biostatistics, Epidemiology and Informatics, Perelman School of Medicine, University of Pennsylvania, Philadelphia, PA, United States; Department of Obstetrics and Gynecology, Perelman School of Medicine, University of Pennsylvania, Philadelphia, PA, United States; Glotech Inc., Bethesda, MD, United States; Department of Laboratory Medicine, Clinical Center, National Institutes of Health, Bethesda, MD, United States; Department of Laboratory Medicine and Pathology, University of Minnesota, Minneapolis, MN, United States; Department of Laboratory Medicine, Clinical Center, National Institutes of Health, Bethesda, MD, United States; Global Center for Asian Women’s Health (GloW), Yong Loo Lin School of Medicine, National University of Singapore, Singapore; Bia-Echo Asia Centre for Reproductive Longevity & Equality (ACRLE), Yong Loo Lin School of Medicine, National University of Singapore, Singapore; Department of Obstetrics and Gynaecology, Yong Loo Lin School of Medicine, National University of Singapore, Singapore

## Abstract

**Background:**

Glycated albumin (GA) has recently been proposed as a screening marker for diabetes among non-pregnant individuals. However, data on GA during pregnancy are sparse and lacking among women of diverse race/ethnicity. We investigated longitudinal concentrations of GA among multiracial pregnant women in the National Institute of Child Health and Human Development (NICHD) Fetal Growth Studies–Singletons.

**Methods:**

We quantified GA and cardiometabolic biomarkers using longitudinal plasma samples collected at 10 to 14, 15 to 26 (fasting), 23 to 31, and 33 to 39 gestational weeks from 214 pregnant women without gestational diabetes. We examined the distribution of GA across pregnancy and its association with participants’ characteristics including race/ethnicity, pre-pregnancy body mass index (ppBMI), and selected cardiometabolic biomarkers. GA trajectories were estimated using a latent class approach.

**Results:**

Medians (interquartile range) of GA concentrations were 12.1% (10.6%–13.4%), 12.5% (10.7%–13.8%), 12.4% (10.9%–13.5%), and 11.5% (10.4%–12.5%) at 10 to 14, 15 to 26, 23 to 31, and 33 to 39 weeks, respectively. There were no significant differences in the pattern among different race/ethnic groups (*P* > 0.53). A minority of women exhibited a GA trajectory characterized by a high concentration of GA at 15 to 26 weeks. GA concentrations were inversely related to ppBMI and plasma low-density lipoprotein and triglyceride concentrations, but were not significantly related to hemoglobin A_1c_, fasting insulin, or glucose over pregnancy.

**Conclusions:**

In this study of individuals who were normoglycemic before pregnancy, plasma GA concentrations stayed relatively constant over pregnancy, decreasing only in late pregnancy. GA concentrations were inversely related to ppBMI and suboptimal lipid profiles, but did not appear to be a sensitive marker for glucose metabolism in pregnancy.

## Introduction

Glycated albumin (GA) has been proposed as a clinical marker of long-term glycemia. Unlike hemoglobin A1c (Hb A_1c_), which is a measure of mean glucose concentrations over the prior 8 to 12 weeks (1), the concentration of GA reflects glycemia over the preceding 2 to 3 weeks (2). Studies in adult populations have generally shown positive correlations of GA with blood glucose and Hb A_1c_ concentrations (3–6), alluding to the potential use of GA as a measure of glycemia, particularly in conditions where Hb A_1c_ concentrations are not readily interpretable, such as during pregnancy (7, 8).

Little is currently known about how GA performs as a clinical marker for glycemia in pregnancy. Of the few studies available, GA concentrations do not appear to correlate well with Hb A_1c_ (7, 9, 10) or blood glucose concentrations (10, 11) in pregnancy; however, how GA relates to other classic measures of glucose metabolism (e.g., insulin concentrations) and maternal cardiometabolic status are unknown. Further, sparse information exists on whether GA differs by maternal characteristics, including ethnicity, and how GA concentrations change across pregnancy in diverse populations given that most studies to date have been conducted in Asian populations (7–12). In this study, we attempted to fill some of these gaps by examining the longitudinal concentrations of GA and exploring the relationship of GA with maternal characteristics, glucose metabolism, and major cardiometabolic biomarkers among a multiethnic cohort of euglycemic pregnant women.

## Materials and Methods

### Study population

The Eunice Kennedy Shriver National Institute of Child Health and Human Development (NICHD) Fetal Growth Studies–Singletons Cohort consisted of 2334 low-risk, pregnant women without obesity (body mass index [BMI] 19.0 to 29.9 kg/m^2^) and 468 pregnant women with obesity (BMI 30.0 to 45.0 kg/m^2^) as defined below (n = 2802 in total) (13).Women from 4 self-identified race/ethnic groups (non-Hispanic White, non-Hispanic Black, Hispanic, and Asian/Pacific Islander) without major pre-existing chronic diseases or medical conditions (including diabetes before pregnancy) were enrolled between gestational weeks 8 to 13 at 12 US clinical centers (2009 to 2013). Longitudinal questionnaire data and biospecimens were collected at enrollment and throughout pregnancy. Medical record abstraction of routine prenatal exam results and delivery discharge diagnoses reports was completed after delivery. Institutional review board approval was obtained for all participating clinical sites, the data coordinating center, and the NICHD. All participants provided written, informed consent.

In a nested gestational diabetes mellitus (GDM) case-control study, 107 women with GDM and 214 women without GDM (controls) were included (14). GDM diagnosis was made by applying the Carpenter and Coustan diagnostic criteria on the oral glucose tolerance test (OGTT) results (15). Two non-GDM controls for each GDM case were matched on maternal age (±2 years), race/ethnicity (non-Hispanic White, non-Hispanic Black, Hispanic, Asian/Pacific Islander), and gestational week of blood collection (±2 weeks) (16). The current analysis used data only from the 214 non-GDM controls.

### Glycated albumin measurement

In the NICHD Fetal Growth Study, blood specimens were collected following a standardized protocol at enrollment (during gestational weeks 8 to 13; non-fasting) and at 3 additional study visits at weeks 16 to 22 (fasting), 24 to 29 (non-fasting), and 34 to 37 (non-fasting). The actual range of the blood collection at each visit ranged from gestational weeks 10 to 14, 15 to 26, 23 to 31, and 33 to 39, respectively. Specimens were immediately processed into EDTA plasma, and the samples were stored below −70°C until analysis. At the 16 to 22 week visit, all women were instructed to fast overnight for 8 to 14 h before their blood samples were drawn.

GA was measured in plasma with the Lucica GA-L enzymatic assay, provided by Asahi Kasei Pharma Corporation, on a cobas 6000 instrument (Roche). Total albumin was measured with bromocresol purple. GA is reported as a percentage of total albumin concentration. The inter-assay coefficient of variation (CV) was 1.6% at 15.6% GA and 1.8% at 35.2% GA. We used guidelines from the Clinical and Laboratory Standards Institute (CLSI) to establish reference intervals.

### Cardiometabolic biomarkers

The following assays were performed on plasma, frozen for ≤12 years, collected at the 4 study visits using Roche Diagnostics assays with an inter-assay CV <4%: glucose (mg/dL), insulin (pmol/L), C-peptide (nmol/L), high sensitivity C-reactive protein (hsCRP; mg/L), high-density lipoprotein (HDL; mg/dL), total cholesterol (mg/dL), and triglycerides (mg/dL). Low-density lipoprotein (LDL, mg/dL) was calculated as: total cholesterol−HDL−(triglycerides/5). Insulin resistance was determined by calculating the homeostatic model assessment for insulin resistance (HOMA-IR): fasting plasma insulin [mU/L]* fasting plasma glucose [mg/dL])/405 (17, 18). Hb A_1c_ (%) was measured in EDTA whole blood using a non-porous ion-exchange HPLC assay (G7 analyzer, Tosoh Bioscience, Inc.) with an inter-assay CV <1.2%. The method is National Glycohemoglobin Standardization Program (NGSP)-certified.

### Other variables of interest

All women underwent a screening ultrasound at enrollment to confirm accurate dating of the pregnancy by last menstrual period, which was then used to calculate gestational weeks at each subsequent visit. At enrollment, women completed detailed questionnaires regarding their medical history and sociodemographic characteristics. Maternal height was measured, and pre-pregnancy weight was self-reported. Pre-pregnancy body mass index (ppBMI; kg/m^2^) was calculated and categorized as normal weight (18.5 to 24.9 kg/m^2^), overweight (25.0 to 29.9 kg/m^2^), or obese (≥30.0 kg/m^2^). Family history of diabetes was classified (yes/no) if a woman's parents or siblings had diabetes.

### Statistical analyses

GA concentrations at each visit were estimated using the median and interquartile range. Differences in the median values were tested across characteristics using linear mixed effects models. The reference intervals (RIs) for GA were derived using a nonparametric approach based on the 2.5th and 97.5th percentiles and corresponding 90% confidence intervals of the population. Spearman correlation was performed to evaluate the relationship between the concentrations of glycated albumin and cardiometabolic biomarkers at each study visit across pregnancy. Correction for multiple testing was performed according to the Benjamini–Hochberg false discovery rate (FDR) method (19). Trajectories of GA were estimated using a flexible data-driven semiparametric latent class approach (20). We compared model fit based on 2 to 4 trajectory groups and fit linear, quadratic, and cubic models. The final model was selected based on maximum Bayesian information criterion value (i.e., least negative). As the analytic sample is composed of the matched non-GDM controls from a nested GDM case-control study, sampling weights were applied to all analyses to return the distribution of demographic characteristics in the sample to baseline distributions for the cohort, as previously described (21–23).

All analyses were performed using SAS version 9.4.

## Results

Women in the cohort were on average 30.3 (standard deviation 5.4) years of age with a mean ppBMI of 25.6 (5.3) kg/m^2^. A total of 24% of women self-reported as Asian/Pacific Islander, 38% as Hispanic, 14% as non-Hispanic Black, and 23% as non-Hispanic White, and 55% were nulliparous (Table 1).

**Table 1.** Baseline characteristics of study participants in the NICHD Fetal Growth Studies–Singletons Cohort, n = 214.

Variable	Mean ± SD or n (%)
Age, maternal, years	30.3 ± 5.4
Race/ethnicity	
Asian/Pacific Islander	52 (24.3%)
Hispanic	82 (38.3%)
Non-Hispanic black	30 (14.0%)
Non-Hispanic white	50 (23.4%)
Pre-pregnancy BMI, kg/m^2^	25.6 ± 5.3
18.5 to 24.9	119 (55.6%)
25.0 to 29.9	61 (28.5%)
≥30.0	34 (15.8%)
Waist-to-hip ratio	0.8 ± 0.1
<0.85	173 (80.8%)
≥0.85	41 (19.2%)
Parity	
0	118 (55.1%)
1+	96 (44.9%)
Family history of diabetes	
Missing	2 (0.9%)
No	164 (76.6%)
Yes	48 (22.4%)
Alcohol consumption 3 months before pregnancy	
No	77 (36.0%)
Yes	137 (64.0%)
Smoking 6 months before pregnancy	
No	213 (99.5%)
Yes	1 (0.5%)
Education	
Less than high school	49 (22.9%)
High school graduate or equivalent	70 (32.7%)
More than high school	95 (44.4%)
Infant sex	
Missing	2 (0.3%)
Male	112 (52.3%)

Median GA concentrations were mostly stable across pregnancy, although a decrease in concentration was observed toward the end of the 3rd trimester; median (interquartile range) GA concentration at gestational weeks 10 to 14 weeks, 15 to 26 weeks, 23 to 31 weeks, 33 to 39 weeks were 12.1% (10.6%–13.4%), 12.5% (10.7%–13.8%), 12.4% (10.9%–13.5%), and 11.5% (10.4%–12.5%), respectively (Table 2). RIs were similar across pregnancy at different study visits, except for a higher upper limit at 15 to 26 weeks; RIs (2.5th and 97.5th percentiles) were 7.8% and 18.5% at gestational weeks 10 to 14 weeks, 8.5% and 38.4% at 15 to 26 weeks, 8.7% and 19.8% at 23 to 31 weeks, and 7.7% and 18.0% at 33 to 39 weeks. No statistically significant differences in GA were observed at any point in pregnancy by women's race/ethnicity, waist-to-hip ratio, infant sex, or the time since the last meal (i.e., fasting duration) (Table 3). Median concentrations tended to be higher among women aged ≥30 years compared to women <30 years; however, the difference was significant only at 33 to 39 weeks (*P* = 0.02). Additionally, GA concentrations tended to decrease with increasing ppBMI; however, the difference was significant only at 23 to 31 weeks (*P* = 0.02).

**Table 2.** Glycated albumin concentrations (median (interquartile range)) and nonparametric reference intervals in pregnancy by study visits, NICHD Fetal Growth Studies–Singletons Cohort.

	Glycated albumin, %			
	10 to 14 weeks (n = 209)	15 to 26 weeks (n = 206)	23 to 31 weeks (n = 203)	33 to 39 weeks (n = 181)
Median (25th, 75th)	12.1 (10.6, 13.4)	12.5 (10.7, 13.8)	12.4 (10.9, 13.5)	11.5 (10.4, 12.5)
2.5th percentile (90% CI)^a^	7.8 (6.3, 8.4)	8.5 (8.1, 9.4)	8.7 (4.7, 9.4)	7.7 (6.6, 8.5)
97.5th percentile (90% CI)	18.5 (17.7, 22.3)	38.4 (35.8, 42.7)	19.8 (17.3, 47.6)	18.0 (16.1, 51.0)

^a^CI, confidence interval.

**Table 3.** Glycated albumin concentrations (median (interquartile range)) over pregnancy by study visits and characteristics, NICHD Fetal Growth Studies–Singletons Cohort.^a^

	Glycated albumin, %			
	10 to 14 weeks (n = 209)	15 to 26 weeks (n = 206)	23 to 31 weeks (n = 203)	33 to 39 weeks (n = 181)
Variable	Median (25th, 75th)	Median (25th, 75th)	Median (25th, 75th)	Median (25th, 75th)
Overall	12.1 (10.6, 13.4)	12.5 (10.7, 13.8)	12.4 (10.9, 13.5)	11.5 (10.4, 12.5)
Age, maternal				
<30 years	11.8 (10.6, 13.2)	12.4 (10.4, 13.5)	12.3 (10.8, 13.3)	11.2 (10.3, 12.1)
≥30 years	12.2 (10.7, 13.5)	12.5 (10.8, 13.9)	12.7 (11.2, 13.5)	11.7 (10.7, 12.8)
* P*	0.36	0.45	0.17	**0.02**
Race/ethnicity				
Asian/Pacific Islander	12.3 (10.9, 13.1)	12.8 (11.2, 13.6)	12.4 (11.4, 13.0)	11.6 (10.7, 12.4)
Hispanic	12.0 (10.4, 13.8)	12.4 (10.9, 13.8)	12.2 (10.8, 13.3)	11.6 (10.7, 12.8)
Non-Hispanic black	12.0 (11.3, 14.1)	12.5 (11.2, 22.1)	12.6 (10.8, 13.9)	11.8 (10.4, 13.1)
Non-Hispanic white	12.0 (10.1, 13.2)	12.0 (10.4, 13.5)	12.6 (11.1, 13.9)	11.4 (9.8, 12.2)
* P*	0.80	0.53	0.81	0.53
Pre-pregnancy BMI, kg/m^2^				
18.5 to 24.9	12.5 (10.9, 13.8)	12.6 (11.0, 13.8)	12.9 (11.6, 13.9)	11.6 (10.7, 12.5)
25.0 to 29.9	11.7 (10.4, 12.9)	12.2 (10.3, 13.5)	12.0 (10.8, 13.3)	11.5 (10.0, 12.8)
≥30.0	11.8 (10.3, 13)	12.0 (10.4, 22.6)	11.6 (10.6, 12.6)	11.4 (10.0, 12.1)
* P*	0.07	0.44	**0.02**	0.40
Waist-to-hip ratio				
<0.85	12.2 (10.9, 13.5)	12.5 (10.7, 13.8)	12.5 (11.1, 13.5)	11.5 (10.4, 12.5)
≥0.85	11.8 (10.3, 13)	12.2 (10.8, 13.6)	12.1 (10.8, 13)	11.6 (10.7, 12.6)
* P*	0.14	0.23	0.10	0.87
Infant sex				
Male	12 (10.5, 13.4)	12.5 (10.9, 13.8)	12.4 (11.2, 13.3)	11.6 (10.7, 12.6)
Female	12.1 (10.7, 13.4)	12.4 (10.7, 13.7)	12.3 (10.9, 13.6)	11.5 (10.4, 12.4)
* P*	0.93	0.88	0.91	0.16
Time since last meal				
0 to 4 h	12.1 (10.5, 13.5)	13.4 (12.6, 13.5)	12.4 (11.1, 13.6)	11.6 (10.5, 12.6)
4 to 8 h	11.8 (10.4, 13)	10.4 (9, 11.2)	12.3 (9.7, 13.1)	11.5 (10.3, 12.5)
8 to 12 h	13.2 (12, 15.5)	12.5 (11, 13.9)	11.9 (10.6, 12.9)	10.8 (9.1, 12.4)
12+ h	12.5 (11.1, 12.9)	12.3 (10.6, 13.7)	11.5 (10.8, 12.3)	11.1 (9.2, 11.9)
* P*	0.50	0.17	0.38	0.56

^a^ *P* value for difference in medians from linear mixed effects models. Significant *P* values are in bold.

**Fig. 1.**
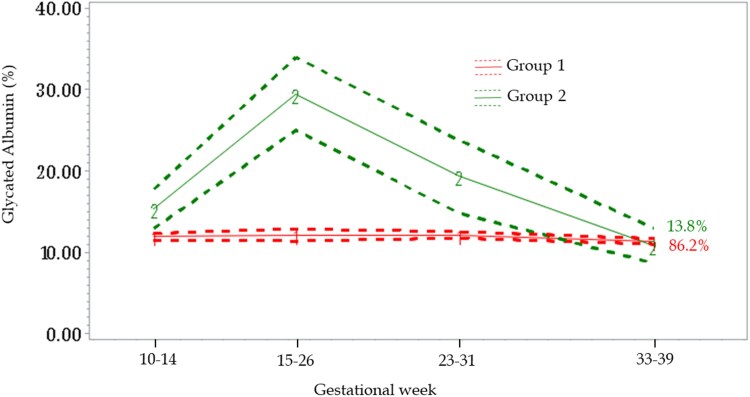
Glycated albumin (GA) trajectories across pregnancy, NICHD Fetal Growth Studies–Singletons Cohort. Trajectories identified based on a semiparametric latent class approach. Group 1 (line “1”) GA stable across pregnancy; Group 2 (line “2”) increase in GA concentrations at the fasting visit. Dotted lines represent 95% confidence intervals. Percentages represent the proportion of data estimated to make up each group.

Latent class trajectories revealed 2 distinct patterns for the longitudinal change in GA across pregnancy. For the majority of women (Group 1: n = 181; 86.2%), GA was stable across pregnancy, however, a small number of women (Group 2: n = 29; 13.8%) experienced an increase in GA concentrations at the fasting visit at 15 to 26 weeks (Fig. 1). Compared to the majority of women (Group 1), a higher proportion of women in the smaller group (Group 2) had a ppBMI ≥25 kg/m^2^, and were of Hispanic ethnicity (32.5% vs 25.2%), non-Hispanic White (36.0% vs 30.4%), or non-Hispanic Black (27.2% vs 23.4%; online Supplemental Table 1).

The correlations between GA and cardiometabolic markers are shown in Table 4. At 15 to 26 weeks gestation, fasting C-peptide concentrations were inversely correlated with GA concentrations (*r* = −0.15; *P* = 0.035). However, no other markers of glucose metabolism were significantly correlated with GA concentrations. At 23 to 31 weeks gestation, C-reactive protein (CRP) concentrations were significantly and inversely correlated with GA concentrations (*r* = −0.21; *P* = 0.032). Additionally, GA tended to be correlated with more favorable lipid profiles (i.e., positively with HDL cholesterol and inversely with LDL cholesterol, total cholesterol, and triglycerides), although the significance and magnitude varied across visits. The number of significant correlations were attenuated after corrections for multiple testing but overall, the results remained similar (online Supplemental Table 2).

**Table 4.** Spearman correlations between the concentrations of glycated albumin and cardiometabolic biomarkers at each study visit across pregnancy, NICHD Fetal Growth Studies–Singletons Cohort.

Cardiometabolic biomarkers	Spearman correlation			
	Glycated albumin, % 10 to 14 weeks n = 209	Glycated albumin, % 15 to 26 weeks n = 206^a^	Glycated albumin, % 23 to 31 weeks n = 107^b^	Glycated albumin, % 33 to 39 weeks n = 102^b^
C-peptide, nmol/L	−0.02	−0.15^c^	−0.05	0.13
HOMA-IR^d^	NA	−0.01	NA	NA
Glucose, mg/dL^e^	0.00	0.01	0.04	0.13
Insulin, pmol/L	0.05	−0.02	−0.06	0.15
C-reactive protein, mg/L	−0.06	−0.04	−0.21^c^	−0.10
Hb A_1c_, %^f^	0.07	−0.05	−0.14	0.03
Cholesterol, mg/dL^g^	−0.04	0.02	−0.07	−0.34^h^
HDL cholesterol, mg/dL^g^	0.16^c^	0.21^i^	−0.16	0.04
LDL cholesterol, mg/dL^g^	−0.05	−0.01	0.03	−0.29^i^
Triglycerides, mg/dL^j^	−0.21^i^	−0.19^i^	−0.08	−0.22^c^

Hb A_1c_, hemoglobin A_1c_; HDL, high-density lipoprotein; HOMA-IR, homeostatic model assessment for insulin resistance; LDL, low-density lipoprotein; NA, not applicable.

^a^Samples collected at 15 to 26 weeks after an overnight fast.

^b^Traditional cardiometabolic biomarkers were only measured in 1 of the 2 controls at visits 2 (23 to 31 weeks) and 4 (33 to 39 weeks).

^c^ *P* < 0.05.

^d^HOMA-IR calculated as fasting plasma insulin [mU/L]*fasting plasma glucose [mg/dL])/405. To convert insulin concentration from pmol/L to mU/L, divide by 6.

^e^To convert glucose concentrations from mg/dL to mmol/L, multiply by 0.0555.

^f^To convert Hb A_1c_ concentrations from % of total hemoglobin to a proportion of total hemoglobin, multiply by 0.01.

^g^To convert cholesterol, HDL cholesterol, and LDL cholesterol concentrations from mg/dL to mmol/L, multiply by 0.0259.

^h^ *P* < 0.001.

^i^ *P* < 0.01.

^j^To convert triglyceride concentrations from mg/dL to mmol/L, multiply by 0.0113.

## Discussion

In this longitudinal study of plasma concentrations of GA among euglycemic pregnant women of multiple races/ethnicities, we observed that plasma GA concentrations stayed relatively constant over pregnancy, with a drop in concentration noticeable only at late pregnancy. GA concentrations tended to be higher among pregnant women older than 30 years and were inversely related to ppBMI, but did not differ by other baseline characteristics, including race/ethnicity or infant sex. GA concentrations were inversely related to suboptimal lipids profiles throughout pregnancy, whereas they did not appear to be a sensitive marker for glucose metabolism in pregnancy.

In line with previous more limited studies, GA concentrations stayed rather constant at the earlier stages of pregnancy, with a decline toward late pregnancy (10, 24–26). Interestingly, we uncovered 2 distinct groups of women in our study based on their longitudinal GA trajectories, where a small group of women (13.8%) exhibited a trajectory characterized by particularly high concentrations of GA at 15 to 26 weeks when a fasting sample was collected. While, in general, fasting duration was unrelated to GA concentrations, we speculate that the higher concentrations could be related to fasting at this time point as it is not explained by any other characteristics of the women.

We are unaware of existing studies that have measured GA concentrations longitudinally among pregnant women of various ethnicities, or estimated GA trajectories across pregnancy. Given that the proportion of women who make up the trajectories—Group 1 and 2—differed significantly by ethnicity and ppBMI, additional studies with larger sample sizes, especially those which include multiethnic pregnant women across the range of ppBMI, are required to further explore the factors contributing to the GA trajectories, and to determine whether differences in these trajectories relate to differences in pregnancy outcomes as well as child health.

Plasma GA concentration was not a sensitive marker of glucose metabolism in pregnancy in this study. GA did not consistently or significantly correlate with glucose or insulin concentrations measured using plasma samples collected at the same time across visits, nor did it correlate with Hb A_1c_, a glycemic biomarker with limited use during pregnancy (8). C-peptide was inversely correlated with GA concentrations at the first 3 visits, but not at the end of pregnancy. We are unaware of published studies that have examined how GA correlates with insulin and C-peptide in pregnant women. In general, our findings are in line with those of previous studies on pregnant women which have examined glucose metabolism using other markers such as Hb A_1c_ and glucose (7, 9–11), suggesting that unlike in nonpregnant populations, plasma GA concentrations are not a useful clinical measure of glycemia in pregnancy. Nevertheless, findings from some studies involving women with GDM suggest that GA concentrations may be linked to infant outcomes, such as birthweight and myocardial hypertrophy (12, 27). More studies are needed to elucidate the mechanisms underpinning the association of GA with infant outcomes.

We found that plasma GA concentrations were related to favorable lipid profiles throughout pregnancy, including lower concentrations of triglycerides and LDL cholesterol. To the best of our knowledge, ours is the first study to examine the relationships between plasma GA and lipids among pregnant women. Similar results were reported by Wang and colleagues when they examined GA concentrations among nonpregnant adults with normal glucose tolerance (28). In that study, the authors observed inverse relationships between GA and triglycerides, total cholesterol, and LDL cholesterol, whilst GA correlated positively with HDL cholesterol (28). The relationship between GA concentrations and plasma lipids appears to differ when comparing participants with or without diabetes (29, 30), suggesting that similar complexity may exist between pregnant populations with or without GDM.

Longitudinal RIs for GA were calculated in this study and they were generally wider than those in published data (9–11, 25, 26); this was particularly evident for the fasting visit at 15 to 26 weeks’ gestation. Longer fasting duration is an unlikely cause of the wider RIs for several reasons. Firstly, GA reflects mean glycemia over 2 to 3 weeks and GA concentrations were shown to be similar in plasma/serum obtained from individuals fasting vs 2 h postprandial (31). Secondly, across the various time points in our study, GA concentrations did not show a clear trend with fasting durations. Some differences in the RIs may be attributed to differences in the assays used to measure GA concentrations (9, 11, 25), which can markedly alter the RI (32); the method used in our study (developed by Asahi Kasei in Japan) is the most widely used method worldwide and the most extensively evaluated method in clinical studies (32). It is important to point out that women included here were the non-GDM controls for a nested case-control study on GDM which included women who were obese, as well as those with conditions that may affect albumin metabolism (33), such as hypothyroidism (14).

Strengths of our study include its prospective and longitudinal nature, with repeated measurements of GA and cardiometabolic biomarkers across multiple time points in pregnancy. Another strength is the inclusion of multiethnic groups of women, including women of Asian/Pacific Islander, Hispanic, non-Hispanic Black, and non-Hispanic White background, making our findings generalizable across these different populations. In addition, we included euglycemic women with varying ppBMI, including women with obesity, as increasingly more women worldwide enter pregnancy with high BMI but may remain euglycemic. For example, >56% of women who had a live birth in the US in 2020 were overweight or obese (34). Lastly, with comprehensive data collected on major sociodemographic and clinical characteristics, we were able to evaluate variations of GA concentrations by these characteristics.

One limitation of this study is that fasting samples were collected at only one of the study visits which may complicate direct comparison of GA across the different visits. However, logistically, it was prohibitively challenging to collect multiple fasting samples from pregnant women. We collected information on time since last meal which enabled us to evaluate how fasting status may affect GA concentrations. We found no clear trend of GA with increasing fasting duration across the visits. Further, as GA concentration reflects glucose control in the past 2 to 3 weeks, its concentration is not sensitive to fasting status. Another potential limitation is that GA assays were performed in samples stored for up to 12 years at −70°C. However, it has been documented, using the same GA assay used in this study, that GA is stable in samples stored under these conditions for up to 23 years (35).

In conclusion, in this study of pregnant women of multi-race/ethnicity, we observed that plasma GA concentrations stayed relatively constant, with a drop in concentration noticeable only at late pregnancy. These findings were consistent across different races/ethnicities. Further, GA concentrations did not correlate well with glycemia markers throughout pregnancy, but higher GA concentrations appear to relate to a favorable lipid profile. Several questions remain to be explored, including how GA relates to pregnancy and infant outcomes given its link with a better lipid profile but not with glycemia.

## Supplemental Material

Supplemental material is available at *Clinical Chemistry* online.

## Supplementary Material

hvad172_Supplementary_Data

## Nonstandard Abbreviations

GA, glycated albumin; NICHD, National Institute of Child Health and Human Development; ppBMI, pre-pregnancy body mass index; Hb A_1c_, hemoglobin A_1c_; GDM, gestational diabetes mellitus; RI, reference interval.
