# Supplementary material for: A Longitudinal Study of Plasma Glycated Albumin across Pregnancy and Associations with Maternal Characteristics and Cardiometabolic Biomarkers
Source: Clin Chem. 2023 Nov 2;69(12):1420–8. doi: 10.1093/clinchem/hvad172 (PMC12376301; doi:10.1093/clinchem/hvad172)
Supplement: hvad172_Supplementary_Data [file hvad172_supplementary_data.docx]

**Supplemental Table 1. Pregnancy characteristics according to glycated albumin trajectory across pregnancy, NICHD Fetal Growth Studies-Singleton Cohort.**

|  | **Glycated Albumin Trajectory Group Across Pregnancy** | | ***p*** |
| --- | --- | --- | --- |
|  | **Group 1 (*n*=181)** | **Group 2 (*n*=29)** |  |
|  | **Mean (SE) or Percent (SE)** | **Mean (SE) or Percent (SE)** |  |
| Age, maternal (years) | 28.0 (0.5) | 27.9 (1.6) | 0.85 |
| Race/ethnicity |  |  | **0.02** |
| Asian/Pacific Islander | 21.1 (3.2) | 4.4 (3.3) |  |
| Hispanic | 25.2 (3.4) | 32.5 (9.6) |  |
| Non-Hispanic Black | 23.4 (4.8) | 27.2 (10.0) |  |
| Non-Hispanic White | 30.4 (4.5) | 36.0 (11.9) |  |
| Pre-pregnancy BMI (kg/m^2^) | 25.3 (0.4) | 26.6 (0.7) | 0.07 |
| 18.5-24.9 | 57.3 (4.5) | 27.8 (9.1) | **0.05** |
| 25.0-29.9 | 29.6 (4.3) | 51.5 (11.2) |  |
| ≥ 30.0 | 13.1 (2.9) | 20.7 (8.0) |  |
| Waist-to-hip ratio | 0.80 (0.0) | 0.80 (0.0) | 0.77 |
| Parity |  |  | 0.17 |
| 0 | 51.3 (4.6) | 34.9 (10.1) |  |
| 1+ | 48.7 (4.6) | 65.2 (10.1) |  |
| Family history of diabetes |  |  | 0.45 |
| No | 79.3 (3.5) | 85.3 (7.3) |  |
| Yes | 20.7 (3.5) | 14.7 (7.3) |  |
| Alcohol consumption 3 months before pregnancy |  |  | 0.41 |
| No | 34.7 (4.3) | 45.2 (11.6) |  |
| Yes | 65.3 (4.3) | 54.8 (11.6) |  |
| Education |  |  | 0.17 |
| Less than high school | 27.5 (4.6) | 12.5 (6.6) |  |
| High school graduate or equivalent | 34.5 (4.3) | 33.2 (11.3) |  |
| More than high school | 38.1 (4.3) | 54.3 (11.4) |  |
| Infant sex |  |  | 0.97 |
| Male | 51.3 (4.4) | 50.9 (11.4) |  |
| Female | 48.7 (4.4) | 49.1 (11.4) |  |
| Fasting duration at visit 0 (hours) | 3.5 (0.2) | 4.6 (1.4) | 0.45 |
| Fasting duration at visit 1 (hours) | 11.7 (0.3) | 12.5 (1.1) | 0.64 |
| Fasting duration at visit 2 (hours) | 2.8 (0.3) | 2.3 (0.6) | 0.81 |
| Fasting duration at visit 4 (hours) | 2.7 (0.3) | 2.0 (0.4) | 0.40 |

Abbreviations: BMI, body mass index.

*p* value for differences between the trajectory groups were estimated using weighted linear regression models or weighted non-parametric models with robust standard errors. Significant *p* values are in bold.

**Supplementary Table 2. Spearman correlations between the concentrations of glycated albumin and cardiometabolic biomarkers at each study visit across pregnancy after correction for multiple testing, NICHD Fetal Growth Studies-Singletons Cohort.**

| Cardiometabolic biomarkers | Spearman correlation^a^ | | | |
| --- | --- | --- | --- | --- |
|  | Glycated Albumin (%)  10-14 weeks *n*=209 | Glycated Albumin (%)  15-26 weeks *n*=206^b^ | Glycated Albumin (%)  23-31 weeks *n*=107^c^ | Glycated Albumin (%)  33-39 weeks *n*=102^c^ |
| C-peptide (nmol/L) | -0.02 | -0.15 | -0.05 | 0.13 |
| HOMA-IR^d^ | NA | -0.01 | NA | NA |
| Glucose (mg/dL)^e^ | 0.00 | 0.01 | 0.04 | 0.13 |
| Insulin (pmol/L) | 0.05 | -0.02 | -0.06 | 0.15 |
| C-reactive protein (mg/L) | -0.06 | -0.04 | -0.21 | -0.10 |
| Hb A_1c_ (%)^f^ | 0.07 | -0.05 | -0.14 | 0.03 |
| Cholesterol (mg/dL)^g^ | -0.04 | 0.02 | -0.07 | -0.34*** |
| HDL cholesterol (mg/dL)^g^ | 0.16 | 0.21** | -0.16 | 0.04 |
| LDL cholesterol (mg/dL)^g^ | -0.05 | -0.01 | 0.03 | -0.29** |
| Triglycerides (mg/dL)^h^ | -0.21** | -0.19** | -0.08 | -0.22 |

Abbreviations: Hb A_1c_, hemoglobin A_1c_; HDL, high-density lipoprotein; HOMA-IR, homeostatic model assessment for insulin resistance; LDL, low-density lipoprotein; NA, not applicable.

^a^ *** *p*<0.001; ** *p*<0.01

^b^ Samples collected at 15-26 weeks after an overnight fast.

^c^ Traditional cardiometabolic biomarkers were only measured in one of the two controls at visits 2 (23-31 weeks) and 4 (33-39 weeks).

^d^ HOMA-IR calculated as fasting plasma insulin [mU/L]*fasting plasma glucose [mg/dL])/405. To convert insulin concentration from pmol/L to mU/L, divide by 6.

^e^ To convert glucose concentrations from mg/dL to mmol/L, multiply by 0.0555.

^f^ To convert Hb A_1c_ concentrations from % of total hemoglobin to a proportion of total hemoglobin, multiply by 0.01.
